# Realization of fully automated quantification of left ventricular volumes and systolic function using transthoracic 3D echocardiography

**DOI:** 10.1186/s12947-017-0121-8

**Published:** 2018-01-23

**Authors:** Lina Sun, Haiyan Feng, Lujia Ni, Hui Wang, Dongmei Gao

**Affiliations:** Department of Ultrasound, China-Japan Union Hospital of Jilin University, 126 Xiantai Street, Changchun, Jilin 130033 China

**Keywords:** 3D echocardiography, Cardiac chamber quantification, Fully automated analysis

## Abstract

**Background:**

Study on automated three-dimensional (3D) quantification of left heart parameters by using Heartmodel software is still in the early stage and fully automatic analysis was not clearly achieved. The aim of our study was to evaluate the performance of this new technology in measuring left ventricular (LV) volume and ejection fraction (EF) in patients with a variety of heart diseases on the basis of rationally determining the default endocardial border values.

**Methods:**

Subjects with a variety of heart diseases were included prospectively. High quality Heartmodel images were selected to determine the end-diastolic and end-systolic default values of endocardial border. The accuracy and reproducibility of automated three-dimensional echocardiography (3DE) for measuring LV end-diastolic volume (EDV), end-systolic volume (ESV) and EF were evaluated with the traditional manual 3DE as the relative standard.

**Results:**

Ninety seven subjects were enrolled in the study. The default endocardial border values were determined as 66% and 40% for end-diastole (ED) and end-systole (ES), respectively. Most of the subjects (84/97) were automatically analyzed by Heartmodel software without manual adjustment, revealing a close correlation of automated 3DE with manual 3DE in measuring EDV, ESV and EF (r-values: EDV: 0.96, ESV: 0.97, EF: 0.96). The EDV and ESV values obtained by automated 3DE were higher than those measured by manual 3DE (biases: EDV: 16 ± 18 ml, ESV: 11 ± 12 ml). The intra- and inter-observer reproducibility of automated 3DE was better than that of manual 3DE. Automated 3DE with manual adjustment showed good consistency with manual 3DE in assessing the impairment degree of systolic function in patients with wall motion abnormalities (*n* = 58), (Kappa = 0.74, *P* = 0.00).

**Conclusion:**

Fully automated 3DE quantification of LV volume and EF could be achieved in most patients. Since automated 3DE was accurate and more reproducible, it could replace the existing manual 3DE technology and be routinely used in clinical practice.

## Background

Accurate measurement of left ventricular (LV) volume and ejection fraction (EF) is essential to determine the prognosis in patients with various heart disease, and consequently helps in establishing treatment decisions as eligibility criteria in many clinical trials [1, 2]. Cardiac magnetic resonance (CMR) is now considered the gold standard for measuring LV volume and EF. However, it is very expensive and cannot be used by bedside patients or patients with implanted devices. Currently, transthoracic echocardiography (TTE) remains to be the technique of choice for imaging. The biplane method of disks summation (modified Simpson’s rule) is the most commonly used technology for measuring LV volume and EF. However, due to technical shortcomings of apex foreshortening and geometrical assumptions, the results are not very satisfactory. Transthoracic three-dimensional echocardiography (3DE) overcomes the above shortcomings. Although volumes tended to be underestimated on 3DE, the accuracy of 3DE was comparable with that of CMR when measuring LV volume and EF [3]. But 3DE, especially traditional manual 3DE, is not widely used in clinical practice due to its time-consuming measurements [4].

In order to apply 3DE technology for routine clinical examination, the newly developed Heartmodel software (HeartModel^A.I.^; Philips Healthcare, Andover, MA, USA) that realizes the rapid and automated three-dimensional (3D) quantitative analysis of LV volume and EF can be used. But the study on automated 3DE technology is still in its early stages due to the following reasons: Firstly, the Heartmodel software detects more robustly the inner and outer extents of the myocardial tissue -- those being at the interface of the blood-tissue and the compacted myocardium, whereas the LV endocardial border between them needs to be subjectively defined by the operator himself. It is well known that the correct endocardial border setting is essential for the accurate measurement of LV volume and EF, which is also the basis for comparing this novel measurement technology with the traditional ones. However, there are still no optimal rationally-determined default endocardial border values that can be routinely used at clinic so far. Secondly, previous studies have reported a great deal of manual editing needed in a large proportion of enrolled subjects following the automated analyses [5–11], which Obviously did not fulfill the function of complete automated measurement for LV volume and EF, hence still has the disadvantage of time consumption. Since one of the reasons might be the lack of reasonable default border values they could refer to, the default endocardial border values should be first determined properly before the evaluation of this new technique, which consequently reduced the manual editing after automated contouring and in turn helped to realize the objective and complete automated quantitative analysis of LV volume and EF. Hence, the purpose of this study was to evaluate the accuracy and reproducibility of automated 3DE technology in measuring LV volume and EF in patients with a variety of heart diseases on the basis of rationally determining the default endocardial border values.

## Methods

### Study design

Patients with a variety of heart diseases were included prospectively. A full-volume image used for manual 3D measurements and two Heartmodel images acquired over a 5 min period were collected in each patient by an experienced physician (DM.G.). Image quality was determined by using a five point scale in which 1, clear display of all 17 segments, well visualization of endocardial trabeculae and clear differentiation from the myocardium in at least 12 segments; 2, visualization of all wall segments and clear differentiation of endocardial trabeculae from the myocardium in 6–12 segments; 3, visualization of all wall segments and clear differentiation of endocardial trabeculae from the myocardium in less than 6 segments; 4, dropout of less than or equal to three segments but visualization of adjacent segments within the same territory; 5, dropout of more than 3 segments. Images in scale 5 were considered poor image quality and excluded from the study. After completing the image acquisition for all subjects, the high quality Heartmodel images in scale 1 were selected for determining the default endocardial border values. Then all Heartmodel images were automated analyzed with the determined default endocardial border values. The time interval for the analysis of two Heartmodel images in each subject should be at least one week and it was applied in a random order. If the automated contouring of endocardial border was not satisfactory, manual editing can be performed. Similarly, each full volume image was analyzed twice by manual 3DE using blinded method. The time required to obtain LV volume and EF with the two methods was recorded. The averaged values of two automated 3D measurements were compared to those from two manual 3D measurements to determine the consistency and differences between the two methods in measuring LV volume and EF. The intra-observer reproducibility of the two methods was also evaluated. Besides, to assess the inter-observer reproducibility of the two methods, 15 subjects were randomly selected and a second dataset was retained by another physician (LN.S.) using the same device in the same place, and the images were analyzed by the same physician (LN.S.). The EF values of patients with wall motion abnormalities was further divided into normal range (male ≥ 52%, female ≥ 54%), mildly abnormal (41–51% for male, 41–53% for female), moderately abnormal (30–40% for both male and female) and severely abnormal (<30% for both male and female) according to the American society of echocardiography and the European association of cardiovascular imaging [12]. Results were compared to evaluate the consistency between automated 3DE and manual 3DE in assessing the degree of impaired systolic function.

### Patients

Between February 2017 and August 2017, 103 patients undergoing TTE in our ultrasound room were prospectively included when there was adequate time for the examination. This study has been approved by the institutional review board and all enrolled subjects have signed the informed consent form. Patients with severe heart malformation or with atrial fibrillation were excluded.

### Automated 3DE

Automated 3DE was performed using the EPIQ system (Philips Medical Systems, Andover, MA, USA) and an X5–1 phased-array transducer with the patient placed in the left lateral decubitus position. After connecting the electrocardiogram, the apical 4-chamber view was displayed with the LV in the center along the volume axis. The X-plane button on the screen was clicked, and the clear imaging of the LV was confirmed on the biplane display. Then the HM ACQ button on the screen was clicked to collect the Heartmodel image. Before acquisition of each image, the images were optimized for endocardial visualization by modifying the gain, compress, and time gain compensation controls. The posture of the patient was adjusted to reduce the shielding of pulmonary gas. Focus was set on the mitral valve -papillary muscle level. The HVR full volume and the Auto SCAN were activated under the HM ACQ mode. Then the stored heartmodel image was called out for automated analysis of LVEDV, LVESV and EF using Heartmodel software.

**Fig. 1:**
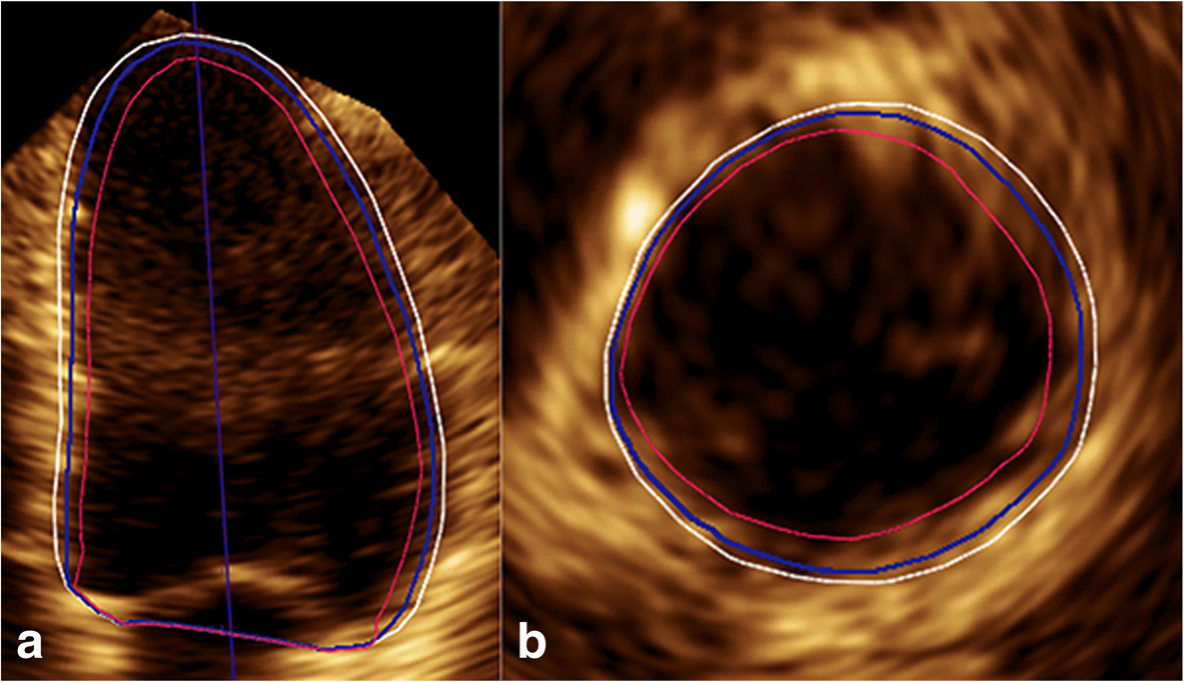
**(a)** LV apical 4-chamber view and (**b**) basal short-axis view in ED showed that the Heartmodel software detected the inner and outer extents of the myocardial tissue, i.e., the blood-tissue interface (red line) and the interface of compacted myocardium (white line), which were assigned to slides of 0% and 100%, respectively. The LV endocardial border (blue line) between them was subjectively defined by the operator, and in this case, it was at the slide of 68%

The mechanism of automated 3DE involves detection of LV endocardial surfaces throughout the cardiac cycle using Heartmodel software, which utilized an adaptive analytics algorithm that consists of knowledge-based identification of initial global shape and orientation followed by patient specific adaptation. As opposed to the detection of single endocardial border, the Heartmodel software detects more robustly the inner and outer extents of the myocardial tissue, i.e., the interface of the blood-tissue and the compacted myocardium. The recognized tissue was divided into 100 slides (0%–100%), where the blood-tissue interface was presented as 0% and the interface of compacted myocardium as 100%. The LV endocardial border between them was subjectively defined by the operator. Taking the measurement of LV volume using CMR as a reference, we included the trabecular muscle in the LV cavity volume [13]. Therefore the endocardial border was defined as the interface between the trabecular muscle and the LV myocardium (Fig. 1). After completing the image acquisition for all subjects, the high quality Heartmodel images were selected for determining the default endocardial border values. With the prerequisite of blinding method on LV volume and EF, the following operations were performed to calculate the default endocardial border values: Firstly, both the ED and ES default endocardial border values were set to 0%; secondly, an experienced physician (DM.G.) adjusted the global ED and ES slides to the closest desired border and recorded the numbers separately. The above operations were repeated on all high quality images, and the ED and ES numbers were added and then the averages were calculated, which were considered as the default endocardial border values and subsequently were applied to all Heartmodel images. If the operator was not satisfied with the automated contouring, manual adjustment was needed, including global and regional editing.

### Manual 3DE

The full-volume images for manual 3D analysis were obtained using the same machine and probe. The images were also obtained from apical 4-chamber view. Full-volume image was required from 4 wedge-shaped subvolumes, which were stitched over 4 consecutive cardiac cycles in a single breath-hold. When capturing the images, the image should be adjusted as in automated 3D to produce the best endocardial visibility. Prototype software QLAB (QLAB 10.5, 3DQ-Advanced, Philips Medical Systems, Bothell, Washington) was adopted for analyzing the full volume images. The 3D volume data were displayed and modified in 3 different cross-sections, which included conventional 2D 4- and 2-chamber views and a short-axis view. The following steps were performed on the end-diastolic and end-systolic frames. First, the users aligned the multiplanar view to maximize the LV cavity long- and short-axes in the 2- and 4-chamber views. Four mitral annular and 1 apical points were then placed on the left ventricle as landmarks in each view. Then, the initial endocardial surface was manually adjusted in multiple apical planes, while including the papillary muscle and endocardial trabeculae in the LV cavity, and its position was corrected as necessary in multiple arbitrary cut planes until the best match was visually verified and the final LVEDV, LVESV, and LVEF values were then recorded.

### Statistics

The correlation between the LV parameters measured by automated 3DE and the ones by manual 3DE was tested using Spearman coefficient. Bland-Altman analysis was used to assess the bias and limits of agreement. Wilcoxon matched paired test was used to verify the significance of the biases. Intra- and inter-observer variability was calculated as the absolute difference of the corresponding pair of repeated measurements as a percentage of their mean in each patient and then averaged over the study group. Kappa test was used to analyze the consistency of categorical data. *P* < 0.05 was considered as statistically significant. Continuous variables are presented as mean ± standard deviation, if normally distributed, or median and interquartile range (IQR) if non-normally distributed; number and percentages are presented for categorical variables.

## Results

Of the 103 subjects included in this study, the image quality of them was classified as scale 1 (*n* = 32 vs *n* = 36), scale 2 (*n* = 13 vs *n* = 17), scale 3 (*n* = 16 vs *n* = 11), scale 4 (*n* = 36 vs *n* = 35) and scale 5 (*n* = 6 vs *n* = 4) with automated 3DE and manual 3DE, respectively. 6 patients were excluded because of poor image quality including 2 patients had poor Heartmodel image quality and 4 patients had poor image quality for both Heartmodel and full-volume. Finally, the study group consisted of 97 subjects, and the 32 subjects who showed high Heartmodel image quality on scale 1 were used to determine the default endocardial border values. The default values of the endocardial border were set at the slides of 66% (66.38 ± 4.56%, range 57%–75%) and 40% (39.78 ± 3.72%, range 33%–47%) for ED and ES, respectively. The baseline demographic and clinical characteristics of all the enrolled subjects and 32 subjects who were used to obtain the default values were shown in Table 1.

**Table 1:** Baseline demographic and clinical characteristics of study subjects

Variables	All subjects (*n* = 97)	Subjects used to determine the default endocardial border values (*n* = 32)
age(y)	52 ± 14	55 ± 13
men	60(62%)	18(56%)
Systolic blood pressure (mmHg)	140 ± 22	140 ± 22
Diastolic blood pressure (mmHg)	87 ± 12	86 ± 12
Heart rate (beats/min)	76 ± 13	72 ± 14
Primary diagnosis		
Coronary heart disease	38(39%)	12(38%)
Valvular heart disease	12(12%)	5(16%)
Hypertensive heart disease	14(15%)	4(12%)
Dilated cardiomyopathy	8(8%)	4(12%)
Hypertrophic cardiomyopathy	3(3%)	0(0%)
Congenital heart disease	4(4%)	0(0%)
No specific heart disease	18(19%)	7(22%)
Frame rate (Hz)		
Automated 3DE datasets	19 ± 3	19 ± 3
Manual 3DE datasets	21 ± 5	23 ± 6

Data are expressed as mean ± SD or as number (percentage)

**Fig. 2:**
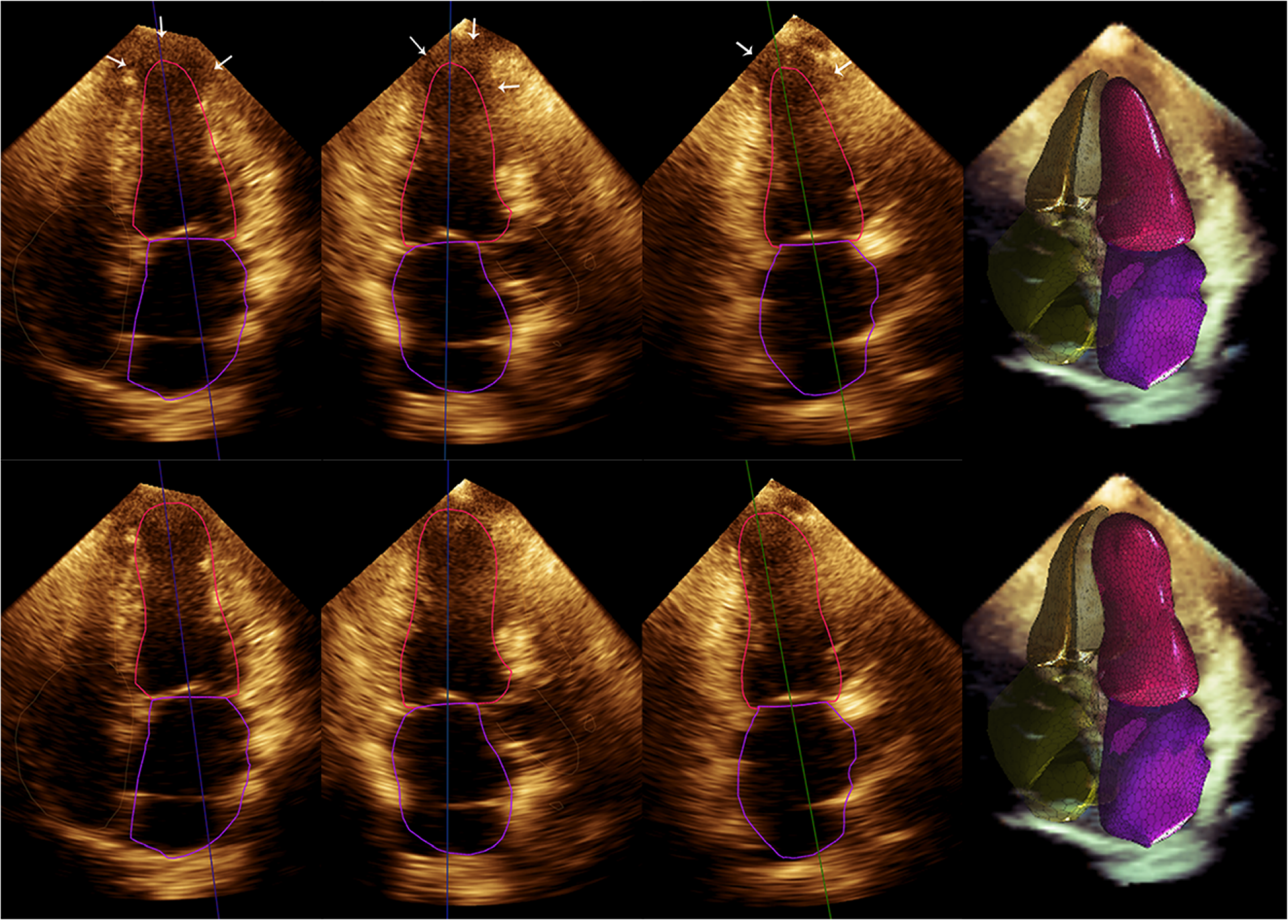
The top line showed the apical segments with regional wall motion abnormality were not correctly recognized (arrow), then manual adjustment was needed (the bottom line)

With the above default border values, 13 subjects required manual adjustment after automated contouring, including 8 cases with apical wall motion abnormalities (Fig. 2), 3 patients with hypertrophic cardiomyopathy and 2 patients with LV wall thickening up to or more than 15 mm due to hypertensive heart disease (*n* = 1) or aortic valve stenosis (*n* = 1). The time required for the LV volume and EF measurements by automated 3DE was 1.12 ± 0.31 min and 3.74 ± 1.62 min for those who did not require manual adjustment and the 13 subjects who needed manual adjustment after automated contouring, respectively, whilst it was 4.93 ± 2.38 min using manual 3DE.

**Fig. 3:**
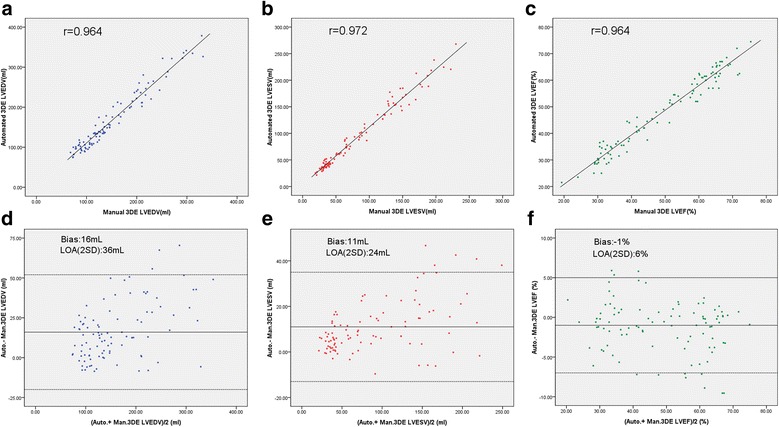
Comparison of LV volume and EF measured by automated 3DE with manual adjustment and manual 3DE. Correlation and Bland-Altman analysis of LVEDV (**a**, **d**), LVESV (**b**, **e**), and LVEF (**c**, **f**). 3DE = 3-dimensional echocardiography; Auto. = automated; Man. = manual; LOA = limits of agreement; LVEDV = left ventricular end-diastolic volume; LVESV = left ventricular end-systolic volume; LVEF = left ventricular ejection fraction

**Fig. 4:**
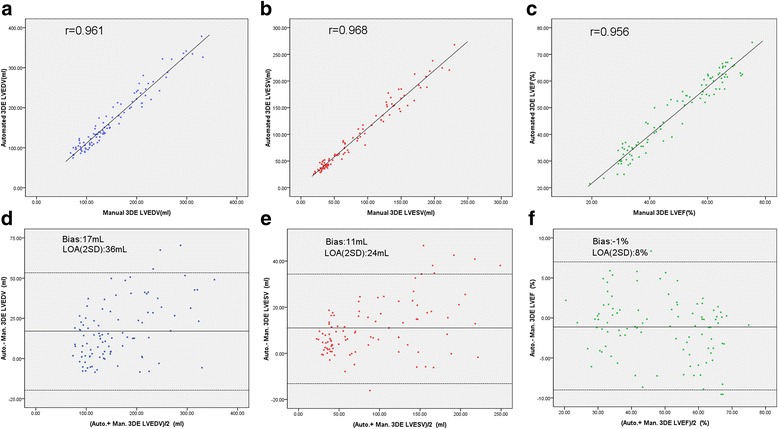
Comparison of LV volume and EF measured by automated 3DE without manual adjustment and manual 3DE. Correlation and Bland-Altman analysis of LVEDV (**a**, **d**), LVESV (**b**, **e**), and LVEF (**c**, **f**). 3DE = 3-dimensional echocardiography; Auto. = automated; Man. = manual; LOA = limits of agreement; LVEDV = left ventricular end-diastolic volume; LVESV = left ventricular end-systolic volume; LVEF = left ventricular ejection fraction

A strong correlation was noted between the automated and the manual 3D measurements of LVEDV, LVESV and LVEF. However, a statistically significant difference was observed between the two methods. Compared with manual 3DE, EDV and ESV obtained by automated 3DE were higher (Table 2, Fig. 3, Fig. 4).

**Table 2:** Comparison of LV volume and EF measured by automated 3DE and manual 3DE

	Automated 3DE measurements	Manual 3DE measurements as relative standard	Correlation (r value)	*P* value	Bias
LVEDV with manual adjustment, ml	143 (108 to 214)	133 (103 to 200)	0.964	0.00	16 ± 18
LVEDV without manual adjustment, ml	148 (109 to 214)	133 (103 to 200)	0.961	0.00	17 ± 18
LVESV with manual adjustment, ml	75 (42 to 152)	66 (36 to 130)	0.972	0.00	11 ± 12
LVESV without manual adjustment, ml	74 (43 to 150)	66 (36 to 130)	0.968	0.00	11 ± 12
LVEF with manual adjustment, %	49 (35 to 61)	51 (34 to 63)	0.964	0.00	−1 ± 3
LVEF without manual adjustment, %	52 (35 to 61)	51 (34 to 63)	0.956	0.00	−1 ± 4

*3 DE* 3-dimensional echocardiography, *Bias* measurement of automated 3DE - measurement of manual 3DE, *LVEDV* left ventricular end-diastolic volume, *LVESV* left ventricular end-systolic volume, *LVEF* left ventricular ejection fraction

Data were expressed as mean ± SD or as median (interquartile range)

The automated 3DE showed better correlation with manual 3DE for classifying the impairment degree of systolic function in patients with wall motion abnormalities (*n* = 58) with manual adjustment (Kappa = 0.74, *P* = 0.00) than without adjustment (Kappa = 0.63, *P* = 0.00). Although there were 10 patients who were assigned to different impairment classification between manual 3DE and automated 3DE with manual adjustment, the EF difference was within 5% in 6 patients. (Table 3).

**Table 3:** Classification of the impairment degree of systolic function in patients with wall motion abnormalities

Automated 3DE	Manual adjustment							
Manual 3DE	A		B		C		D	
	Yes	No	Yes	No	Yes	No	Yes	No
A	5	6	2	1	0	0	0	0
B	1	3	13	10	1	2	0	0
C	0	0	2	2	24	24	4	4
D	0	0	0	0	0	2	6	4

*3DE* 3-dimensional echocardiography

A = normal range (male ≥ 52%,female ≥ 54%); B = mildly abnormal (male 41–51%,female 41–53%);C = moderately abnormal (30–40% for both male and female); D = severely abnormal (<30% for both male and female)

The results of intra- and inter-observer variability were shown in Table 4. Intra- and inter-observer reproducibility of automated 3DE were better than those of manual 3DE.

**Table 4:** Intra- and inter-observer variability comparison

Variability (%)	Manual 3DE			Automated 3DE with manual adjustment			Automated 3DE without manual adjustment		
	EDV	ESV	EF	EDV	ESV	EF	EDV	ESV	EF
Intra-observer	6 ± 4	9 ± 7	8 ± 5	5 ± 4	6 ± 5	6 ± 5	5 ± 4	6 ± 4	6 ± 5
Inter-observer	8 ± 5	10 ± 7	7 ± 3	5 ± 4	8 ± 5	7 ± 4	5 ± 4	8 ± 5	7 ± 4

*3DE* 3-dimensional echocardiography, *EDV* end-diastolic volume, *ESV* end-systolic volume, *EF* ejection fraction

## Discussion

This study showed that the automated 3DE measurement, assisted with few manually adjusted Heartmodel images (13/97), was highly accurate in measuring LV volume and EF in patients with a variety of heart diseases. In addition, it was more timesaving and reproducible than the manual 3DE. The LVEDV and LVESV values obtained by automated 3DE were higher compared to manual 3DE, and there was a good consistency in assessing the impairment degree of systolic function in patients with wall motion abnormalities with these two technologies.

The Heartmodel software was designed to automatically measure LV volume and EF in order to reduce subjective factors, increase reproducibility and save time. However, previous studies on automated 3DE demonstrated a large amount of manual editing following after the automated contouring [5–11]. The purpose of fully automated measurement was therefore not clearly achieved, and the advantages of the new technology were not presented totally. Our study tended to realize the automated measurement to the greatest extent by rationally setting the default endocardial border values. Among the 97 enrolled patients, only 13 images required manual editing. Special attention should be paid to the fact that all the 8 patients with apical wall motion abnormalities were incorrectly automated contoured (Fig. 2). The reason may be that the apical myocardium located in the near field of ultrasound was not clear in apical 4-chamber view because of reverberation artifact and focus location which was set at the mitral valve-papillary muscle level. This in turn affected the recognition and tracking of the endocardium in the apex region by Heartmodel software. We suppose that the wall motion of apical segments was approximately estimated on the basis of basal and midventricular segment wall motion. Our conjecture was supported by the following phenomena: for patients with global wall motion abnormalities or without wall motion abnormalities, the software can correctly outline the apical endocardium because the amplitude of LV wall motion in these cases was essentially the same in all segments, whilst for patients with regional wall motion abnormalities in the apical segments, the Heartmodel software seemed to outline the apical endocardium according to the basal and midventricular segment wall motion instead of tracing the true position of apical endocardium. Therefore, when apical wall motion was inconsistent with the basal and midventricular segment motion, attention should be paid whether manual adjustment was needed in apical segments. The other 5 patients who needed manual adjustment consisted of 3 patients with hypertrophic cardiomyopathy and 2 patients with prominent LV wall thickening due to hypertensive heart disease or aortic valve stenosis. Uneven wall thickening and hypoechoic myocardium close to the endocardium accounted for the inaccurate automated contouring. According to our study, fully automated quantification of LV volumes and systolic function using Heartmodel software could be achieved in most patients and manual adjustment was needed merely in the minority of the patients.

In this study, the default endocardial border values were set as 66% and 40% for ED and ES, respectively. EDV and ESV obtained by automated 3DE were higher compared to manual 3DE, and the bias for EDV and ESV were 16 ± 18 ml and 11 ± 12 ml, respectively. The different methods of determining the endocardial border with the automated and manual 3DE accounted for the biases: On automated 3DE, although the endocardial border cannot be clearly explored in most patients, the more robustly recognized inner and outer extents of the myocardial tissue, i.e., the interface of the blood-tissue and the compacted myocardium can be easily detected by Heartmodel software. In our study we obtained the default endocardial border values from high quality heartmodel images, which represented the relative position of the endocardial border between the inner and outer extents of the myocardial tissue with varying heart shapes and sizes. Then all the subsequent Heartmodel images were automated analyzed with the determined default border values. So technically we could count trabeculae muscle in the LV volume on automated 3DE regardless of the image quality. On the contrary, on manual 3DE, although the trabeculae was planned to be counted in LV volume, the spatial resolution in most patients was insufficient to clearly define the endocardial trabeculae, which was, as a result, lumped together with the myocardium rather than being included in the LV cavity. This was the most significant potential source of volume underestimation by 3DE [14]. Most previous studies but two [6, 11] supported that the LV volumes obtained by automated 3DE were higher than those by manual 3DE [5, 8–10]. In addition, automated 3DE and CMR were compared in three studies, and results showed that the LV volumes were still underestimated by automated 3DE [7, 8, 11]. A meta-analysis of 34 studies reported that the overall pooled biases of manual 3DE were −19.1 ± 17.1 ml and −10.1 ± 14.9 ml for EDV and ESV compared with CMR [3]. According to our results, the measured values using automated 3DE might be very close to that of CMR values. A multicentric study also came up with the result that the automated 3DE measurements were closer to that of CMR than manual 3DE [15].

Practically, EF is the most important index in assessing the cardiac function for clinicians. Previous studies have reported that automated 3DE and manual 3DE were highly consistent in measuring EF. While some studies suggested no significant difference between the two methods [5, 6, 11], two studies found that the EF measured by automated 3DE was slightly lower than that measured by manual 3DE [8, 10]. Our study arrived at similar result with the bias −1%, which might be explained by the fact that the frame rate of full volume image was slightly higher than that of Heartmodel images. The impairment degree of EF in patients with wall motion abnormalities was further classified in the present study and the results showed that the automated 3DE with manual adjustment showed better correlation with manual 3DE than that without adjustment. Without manual adjustment, automated 3DE tended to overestimate the EF measurement in patients with regional kinetic abnormalities of the apex and underestimate it in patients with prominent LV wall thickening.

Manual 3DE has been considered the most reproducible ultrasonic technology for measuring LV volume and EF so far [16]. According to our results, the reproducibility among inter- and intra-observer using automated 3DE was better than those using manual 3DE in measuring EDV, ESV and EF. This was consistent with a multicentric validation study describing both inter- and intra-observer variability were lower for the automated measurements than conventional manual technology for all parameters [6]. The lower variability of the automated method might be explained by the fact that the automated 3DE measurement did not require much experience to operate and automated analytic method was more objective.

This study has some limitations. Firstly, CMR was currently recognized as the gold standard for measuring LV volume and EF; however, manual 3DE rather than CMR was used as a reference standard in our study. Although the volume tended to be underestimated, the accuracy of manual 3DE was proved to be comparable with that of CMR [3]. In order to warrant the accuracy as standard, all of full-volume images for manual 3DE were analyzed by an experienced physician. Secondly, our sample size was not large enough to include all kinds of heart diseases, and further research is still needed to improve the results.

## Conclusion

Automated 3DE could measure LV volume and EF accurately and fully automated quantification could be achieved in most patients. Since it was timesaving and more reproducible, automated 3DE could replace the existing manual 3D technology and be routinely used in clinical practice.

## Abbreviations

3-D: Three-dimensional
3DE: Three-dimensional echocardiography
CMR: Cardiac magnetic resonance
ED: End-diastole
EDV: End-diastolic volume
EF: Ejection fraction
ES: End-systole
ESV: End-systolic volume
IQR: Interquartile range
LV: Left ventricular
TTE: Transthoracic echocardiography
